# A longitudinal perspective on childhood adversities and onset risk of various psychiatric disorders

**DOI:** 10.1007/s00787-014-0540-0

**Published:** 2014-04-11

**Authors:** Albertine J. Oldehinkel, Johan Ormel

**Affiliations:** Interdisciplinary Center Psychopathology and Emotion Regulation, University of Groningen, University Medical Center Groningen, CC 72, P.O. Box 30001, 9700 RB Groningen, The Netherlands

**Keywords:** Childhood adversity, Adolescents, Depression, Anxiety, Disruptive behavior

## Abstract

It is well-known that childhood adversities can have long-term effects on mental health, but a lot remains to be learned about the risk they bring about for a first onset of various psychiatric disorders, and how this risk develops over time. In the present study, which was based on a Dutch longitudinal population survey of adolescents TRAILS (*N* = 1,584), we investigated whether and how childhood adversities, as assessed with three different measures, affected the risk of developing an incident depressive, anxiety, or disruptive behavior in childhood and adolescence. In addition, we tested gender differences in any of the effects under study. The results indicated that depressive, anxiety and disruptive behavior disorders each had their own, characteristic, pattern of associations with childhood adversities across childhood and adolescence, which was maintained after adjustment for comorbid disorders. For depressive disorders, the overall pattern suggested a high excess risk of incidence during childhood, which decreased during adolescence. Anxiety disorders were characterized by a moderately increased incident risk during childhood, which remained approximately stable over time. Disruptive behavior disorders took an intermediate position. Of the three childhood adversities tested, an overall rating of the stressfulness of the childhood appeared to predict onset of psychiatric disorders best. To conclude, the risk of developing a psychiatric disorder after exposure to adversities early in life depends on the nature of the adversities, the nature of the outcome, and the time that has passed since the adversities without disorder onset.

## Introduction

Many studies have revealed associations between childhood adversities and mental health problems later in life, suggesting that these adversities often have long-term effects on functioning and well-being [1–5]. Yet, the mere existence of such an association is not very informative about the chain of events linking the adversities experienced as a child to psychopathology during adulthood [6, 7]. The existing evidence has made clear that this chain involves a highly complex interplay of multiple personal and environmental factors. In a recently published extensive review on childhood determinants of adult psychiatric disorder [8], Fryers and Brugha stated that “Definitions of childhood adversity are truly problematic: they inevitably overlap with apparently more specific variables such as negative life events, child abuse or family conflict, and must be expected to inter-relate with variables such as divorce of parents, child behaviour and child psychological disturbance.” (p.3). A further complicating factors concerns the large time lag between childhood and adulthood; the longer the period between determinant and outcome, the larger the number of possible intermediate pathways. Investigating the mental health consequences of childhood adversities during the intermediate period, adolescence, may help to elucidate the link between childhood adversities and adult mental health problems, and to finetune the risk associated with childhood adversities in a dynamic way. The more is known about the short-term and longer-term aftermath of childhood adversities, the better we will be able to estimate the prognosis of an individual with a history of childhood adversities, and to adjust and improve the accuracy of the prognosis over time. Longitudinal surveys offer excellent opportunities to better understand and predict the development of mental health problems after exposure to childhood adversities [8]. This article describes an attempt to do so based on a Dutch longitudinal population survey of adolescents, TRAILS.

That childhood adversities are associated with an increased risk to have mental health problems in adolescence and adulthood does not necessarily imply that they are associated with an equally increased risk to develop such problems later in life. The few studies that accounted for problem levels in the period in-between suggest that this may not always be true. Appleyard et al. [9] found that the association between maternal support in early childhood and internalizing problems at age 16 was mediated by internalizing problems at school entry. Consistent with that, a recent study in our own cohort TRAILS indicated that the effect of childhood family instability on internalizing and externalizing problems in late adolescence was mediated by early-onset mental health problems rather than continued family instability [10].

These prior findings provide a rationale to hypothesize that the risk of an incident onset of psychiatric disorder after exposure to childhood adversities attenuates over time. Indeed, in the US National Comorbidity Survey (NCS), associations of childhood adversities with first onsets of psychiatric disorders declined with lifecourse stage [11]; a finding that was replicated in the World Mental Health Survey and NCS Replication survey [7, 12]. A drawback of these studies is that they included respondents with a wide age range, implying that both the childhood adversities and the onset of psychiatric disorders could have occurred a long time ago, which provides a potential source of recall bias [12, 13]. The TRAILS study allowed to reduce the time lag between the occurrence of these events and their measurement and to use multiple informants, which will enhance the validity of the measures.

The present study was conducted to test the hypothesis of decreasing effects of childhood adversities over time by means of a Cox proportional hazards regression model with separate risk estimates for childhood and adolescence, that is, during and after exposure to the childhood adversities. Specific aims were: (1) to investigate similarities and differences between three major groups of disorders: depressive disorders, anxiety disorders, and disruptive disorders; (2) to compare the effects of different kinds of childhood adversities; and (3) to explore gender differences in any of the effects.

The aim to investigate similarities and differences between depressive, anxiety, and disruptive behavior disorders was inspired by the fact that these three groups of disorders often occur in concert and have all been related to childhood adversities [3, 6, 7, 12, 14], but are also assumed to have partly diverging etiologies [15–17] and associated endophenotypes (e.g., [18, 19]). In the Adolescent Supplement of the National Comorbidity Survey, childhood adversities were found to predict disruptive behavior disorders most strongly and anxiety disorders least strongly [3]. Although these findings do not provide a solid basis for specific hypotheses with regard to time-dependent effects of childhood adversities, they do indicate that differential effects are well conceivable.

We examined the effect of various measures and types of adversities because, although different kinds of childhood adversities tend to co-occur [3, 7, 20], some adversities have been found to be stronger associated with later mental health outcomes than others (e.g., [3, 7, 8, 12]). Only by studying a variety of childhood adversities in the same sample can we make comparative statements about their impact in the short and longer term.

Gender differences were explored because of various indications that the short-term and long-term effects of childhood adversities might be different for boys and girls. To start with, the prevalence of these disorders is known to be different for boys and girls, with boys being overrepresented in the disruptive behavior disorders, and girls in anxiety and depressive disorders (e.g., [21]). Furthermore, Rutter and colleagues [22] pointed to the fact that boys tend to predominate in neurodevelopmental disorders with an early onset, and girls in emotional disorders with an adolescent onset, and related these differences to genetic gender differences, the biological and psychosocial consequences of these differences, and gender differences in the distribution of more proximal risk factors. Not only the risk of disorder itself, but also the sensitivity to interpersonal stressors might develop differentially for boys and girls over time [23–25], possibly as a result of differential actions of male and female sex hormones [26]. Cyranowski et al. [27] provided a theoretical model for these developmental differences by postulating that girls become increasingly sensitive to interpersonal stressors during adolescence, due to a combination of puberty-related changes in oxytocin levels and social pressures to behave in a feminine way. In the context of the present study, it is particularly relevant that this increased sensitivity occurs mainly in at-risk girls, that is, girls with pre-existing vulnerabilities, according to the model, and that childhood adversities are a likely cause of such vulnerabilities. These notions might imply that the assumed decrease in the onset risk associated with childhood adversities in adolescence is stronger in boys than in girls, or even that girls experience an increase rather than a decrease. In a prior study, we found that parental divorce in childhood was associated with an increase in girls’, but not boys’, depressive symptoms during early adolescence [28], suggesting that adolescence is a period that can enhance preexisting susceptibilities in a gender-specific way indeed.

To sum up, we aimed to investigate whether and how childhood adversities, as assessed with a variety of different measures, affected the risk of developing an incident psychiatric disorder in childhood and adolescence, with the expectation that it would decrease over time. In addition to an assessment of overall risk, we examined depressive, anxiety, and disruptive behavior disorders separately, as well as gender differences in any of the effects under study. More knowledge about the dynamics of the impact of childhood adversities on the risk of onset of psychiatric disorders, or their non-occurrence, can help to improve prediction models, and allow them to be extended and refined continuously, with every increasing year.

## Methods

### Sample and procedure

The data were collected as part of the TRacking Adolescents’ Individual Lives Survey (TRAILS), a prospective cohort study of Dutch adolescents with bi-measurements or triennial measurements from age 11 onwards [29, 30]. Participants were recruited from five municipalities in the North of The Netherlands, including both urban and rural areas. Four assessment waves have been completed to date; the present study was based on data from the first, second, and fourth wave (T1, T2 and T4). T1 ran from March 2001 to July 2002, T2 from September 2003 to December 2004, and T4 from October 2008 to September 2010. At T1, 2,230 participants were enrolled in the study (response rate 76 %, mean age 11.1, SD = 0.6, 51 % girls [31]), of whom 96 % (*N* = 2,149, mean age 13.6, SD = 0.5, 51 % girls) participated at T2 and over 83 % (*N* = 1,881, mean age 19.1, SD = 0.6, 52 % girls) again at T4. Of all T4 participants, 84 % (*N* = 1,584) agreed to have a diagnostic interview. The North of The Netherlands has relatively few immigrants: only 10.6 % of the TRAILS cohort originated from a non-western country (mostly Morocco, Turkey, Surinam, the Dutch Antilles, and Indonesia). Furthermore, 15.5 % of the children were raised in single-parent families. Most families (78.0 %) included two or three children. At T1, 16.8 % of the TRAILS participants lived in families with a disposable annual income up to € 13.620, which is below the at-risk-of-poverty-threshold. The study was approved by the Dutch Central Committee on Research Involving Human Subjects (CCMO). Participants were treated in compliance with APA ethical standards and the Declaration of Helsinki, and all measurements were carried out with their adequate understanding and written consent.

### Measures

#### Childhood adversities

Exposure to childhood adversities was operationalized in three different, not mutually exclusive, ways. The first measure, labeled ‘Stress rating’, involved an overall rating of the stressfulness of the participant’s life during childhood. At T2, we assessed parent-reported and self-reported ratings of overall stressfulness of the child’s life between the ages 0–5 and 6–11, respectively. Parents were asked ‘How stressful was your child’s life in this life phase?’, and the adolescents ‘How many bad things happened to you in this period?’ The stressfulness was rated on an 11-point scale, ranging from 0 = not at all to 10 = very much. The parent-reported and self-reported ratings were moderately correlated with each other (*r* = 0.28), and averaged in order to construct an overall multi-informant rating for the period from age 0 to age 11. The relatively low correlation indicates shared variance, but also a considerable unique component in each informant’s rating. Nevertheless, parent-reported and self-reported stress levels were similarly associated with future depressive episodes and stress-reactivity [32], and therefore we felt justified to combine them into a single measure. The second measure, labeled ‘Event index’, reflected the occurrence of a number of specific major events, which were assessed as part of an interview with one of the parents at T1. The events included were death of a family member or other beloved one, parental divorce, and long (>3 months) absence from home. Death of a father, mother or sibling had severity score 3, parental divorce and absence from home score 2, and death of another beloved one (usually a grandparent) score 1. These scores were summed into a severity index. The third measure, ‘Sexual abuse’, was assessed at T4, based on a list of four unwanted sexual acts by an adult family member, friend of the family or stranger, ranging from touching to sexual intercourse, the occurrence of which could be rated as 1 = never happened to me, 2 = happened once, or 3 = happened several times. Events were only included if they had occurred before the age of 12. The sexual abuse measure reflects the mean of the four ratings. Descriptive statistics of the childhood adversities are given in Table 1. Please note that all measures reflect adversities during the period up until 11 years.

**Table 1:** Descriptive statistics

	Mean (SD) or (%)		
	Total group (*N* = 1,584)	Boys (*N* = 728)	Girls (*N* = 856)
Childhood adversities			
Stress rating, age 0–11 (range 0–10)	2.03 (1.58)	2.08 (1.54)	1.99 (1.62)
Events index, age 0–11 (range 0–8)	1.05 (1.05)	1.01 (1.06)	1.08 (1.06)
Sexual abuse, age 0–11 (range 1–3)	1.02 (0.16)	1.01 (0.13)	1.03 (0.18)
Psychiatric outcomes (lifetime prevalence)			
Depressive disorders	21.3 %	13.9 %	27.7 %
Anxiety disorders	26.5 %	18.8 %	32.9 %
Disruptive behavior disorders	13.3 %	16.3 %	10.7 %

#### Psychiatric disorders

During the fourth assessment wave, psychiatric disorders were assessed by means of the World Health Organization Composite International Diagnostic Interview (CIDI), version 3.0. The CIDI is a structured diagnostic interview, which yields lifetime and current diagnoses according to the definitions and criteria of the Diagnostic and Statistical Manual of Mental Disorders (DSM-IV). The CIDI has been used in a large number of surveys worldwide [33], and shown to have good concordance with clinical diagnoses [34, 35]. In addition to the occurrence of psychiatric disorders, the CIDI yields their age of onset. The present study used data on onsets of depressive, anxiety, and disruptive behavior disorders. Depressive disorder was operationalized as the occurrence a major or minor depressive episode or dysthymia. Anxiety disorder was defined as a diagnosis of agoraphobia, generalized anxiety, panic disorder, separation anxiety, social phobia, or specific phobia. Disruptive behavior disorders included oppositional defiant and conduct disorders. Age of onset refers to the age these disorders emerged for the first time. In case adolescents had multiple depressive, anxiety or disruptive behavior disorders, we used the age of the earliest onset. The lifetime prevalences of these disorders are presented in Table 1.

### Statistical analysis

To describe the prevalence and age at onset of the outcome variables in this sample, we calculated their lifetime prevalence per age, separately for boys and girls, and plotted both the absolute prevalence (expressed in percentage of the total sample) and the relative age at onset, that is, the probability of having experienced an onset at a specific age given a lifetime diagnosis of the disorder. After that, we examined whether the three childhood adversity measures were associated with the onset of, respectively, a depressive disorder, an anxiety disorder, and a disruptive behavior disorder, using a Cox proportional hazards regression model with age of first onset as dependent variable and the childhood adversity measure as predictor. In addition to the main effect of the childhood adversity measure we included its interaction with a time-dependent variable indicating the period after childhood, that is, from age 12 onwards. The interaction with this time-dependent variable reflects the difference in the effect of childhood adversities between adolescence (12 years and older) and childhood (0–11 years, the reference period). We chose to model time-dependent effects by only two periods because the low prevalence of some childhood adversities (notably sexual abuse) combined with the relatively low number of new onsets for disruptive disorders during adolescence did not permit a more fine-grained analysis. Gender was included as a covariate in all models. The effects were estimated both with and without adjusting for comorbid disorders. Comorbid disorders were included as time-dependent covariates, implying that they were only taken into account if they had an earlier onset than the outcome disorder. In addition to estimating the effect of each of the childhood adversity measures separately, we also fitted a model that included all three measures simultaneously. In a second step, we tested if the effects of childhood adversities, or their time-dependence, differed between boys and girls by including interactions with gender. To ease interpretation and comparison of the regression coefficients, all continuous variables were standardized to mean 0 and standard deviation 1. The effects of childhood adversities are expressed in hazard ratios, that is, the exponent of the regression coefficients (HR = *e* ^B^), with regard to both childhood and adolescence. The analyses were performed using SPSS version 20. The study had a descriptive rather than strictly hypothesis-testing nature, that is, we did not use *p* values to decide whether or not to reject any null hypotheses, but a *p* value of 0.05 or less was considered an indication that the effect found might reflect an association in the population.

## Results

### Descriptive statistics

**Fig. 1:**
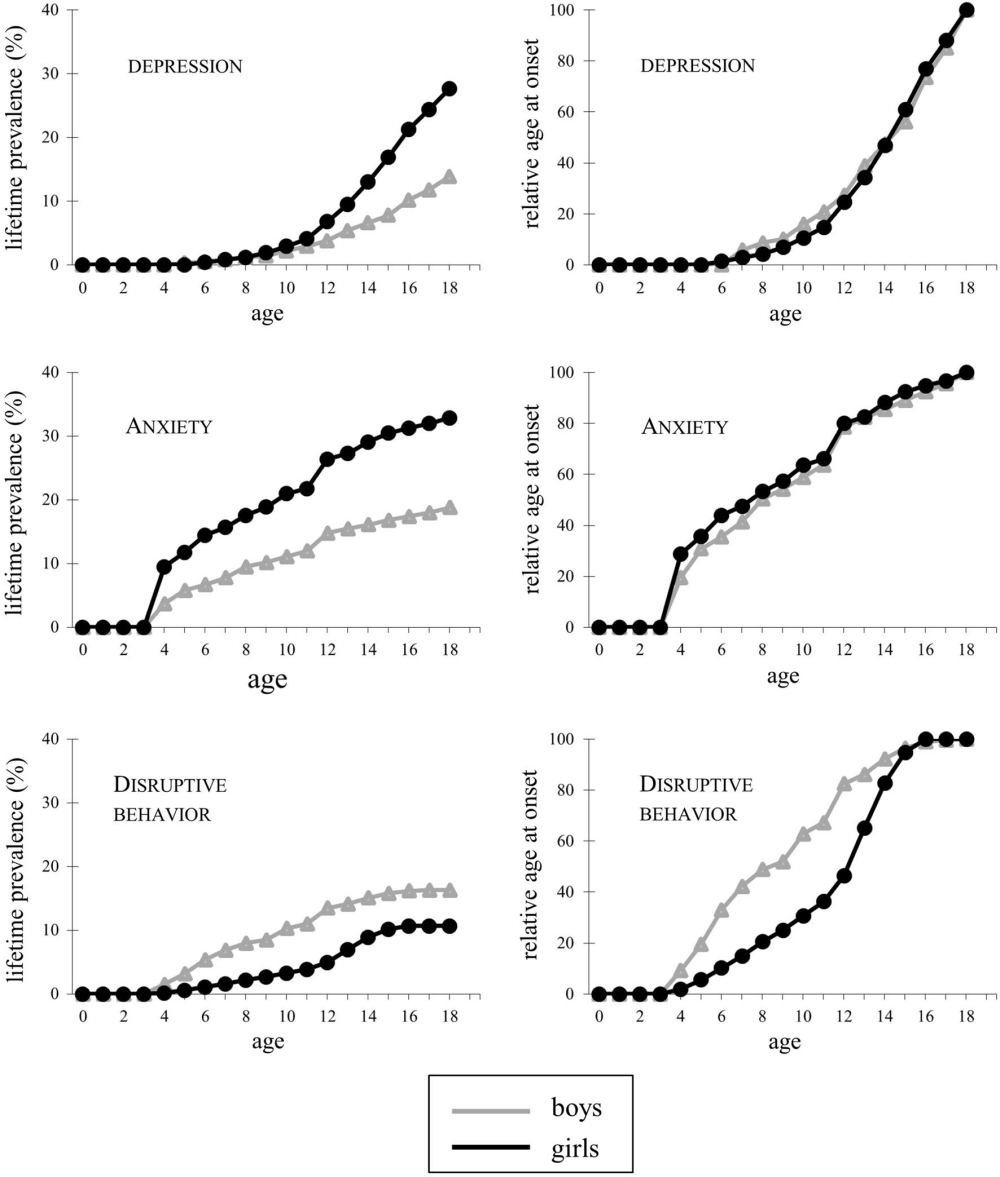
Lifetime prevalences and relative age at onsets for, respectively, depressive disorders, anxiety disorders, and disruptive behavior disorders, by gender. Note: The relative age at onset represents the probability of having experienced an onset at a specific age given a lifetime diagnosis of the disorder

As the mean scores on the three child adversity measures show, the majority of the sample had experienced low adversity levels during childhood. Sexual abuse was particularly rare; only 2.2 % of the sample scored above the lowest possible value of 1. The three adversity measures were significantly, but weakly, associated with each other, with correlations ranging from *r* = 0.05 (*p* = 0.03) for the association between the event index and sexual abuse to *r* = 0.28 (*p* < 0.001) for the association between the overall stress rating and the event index. Consistent with prior research, depressive and anxiety disorders were more prevalent in the girls, and disruptive behavior disorder in the boys in TRAILS (Table 1). Figure 1 shows that, on average, anxiety and disruptive behavior disorders had an earlier onset than depressive disorders. It is further interesting to note that, despite substantial differences in the lifetime prevalence of depression and anxiety, the onset pattern (i.e. relative age at onset) was remarkably similar for boys and girls. This was not true for disruptive behavior disorders, which relatively often had a childhood onset in boys and an adolescence onset in girls.

### Effect of childhood adversities on risk of onset of psychiatric disorder

Table 2 presents the effects of the childhood adversities measures on risk of onset during childhood (age 0–11 years) and adolescence (age 12–19), as well as the significance of the difference between childhood and adolescence risk, for the three different psychiatric outcomes. The majority of the associations tested indicated that all childhood adversity measures were associated with an increased risk of onset of psychiatric disorder, and that this risk pertained to all three disorders under study. Overall, childhood adversities tended to be most strongly associated with depressive and least strongly with anxiety disorders during childhood, while the differences between the three outcomes had largely disappeared during adolescence.

**Table 2:** Associations between childhood adversities and risk of onset of psychiatric disorders in childhood and adolescence, based on Cox regression models, expressed in hazard ratios (HRs)

	Depressive disorder		Anxiety disorder		Disruptive behavior	
	HR	*p*	HR	*p*	HR	*p*
*Unadjusted for comorbity*						
Stress rating						
Childhood (0–11)	1.80	<0.001	1.35	<0.001	1.68	<0.001
Adolescence (12+)	1.32**	<0.001	1.37	<0.001	1.45	<0.001
Event index						
Childhood (0–11)	1.42	<0.01	1.10	0.10	1.23	<0.05
Adolescence (12+)	1.08*	0.17	1.11	0.20	1.23	<0.05
Sexual abuse						
Childhood (0–11)	1.23	<0.001	1.11	<0.01	1.04	0.67
Adolescence (12+)	1.17	<0.001	1.11	0.13	1.16	<0.01
*Adjusted for comorbidity*						
Stress rating						
Childhood (0–11)	1.66	<0.001	1.32	<0.001	1.64	<0.001
Adolescence (12+)	1.17**	<0.01	1.24	<0.01	1.36	<0.01
Depressive disorder			2.43	<0.001	2.15	<0.01
Anxiety disorder	2.23	<0.001			1.93	<0.001
Disruptive behavior disorder	2.06	<0.001	1.87	<0.01		
Event index						
Childhood (0–11)	1.36	<0.01	1.09	0.14	1.20	<0.05
Adolescence (12+)	1.02*	0.70	1.05	0.55	1.18	<0.05
Depressive disorder			2.87	<0.001	2.34	<0.001
Anxiety disorder	2.39	<0.001			2.14	<0.001
Disruptive behavior disorder	2.32	<0.001	2.21	<0.001		
Sexual abuse						
Childhood (0–11)	1.19	<0.001	1.10	<0.05	1.01	0.88
Adolescence (12+)	1.13	<0.01	1.03	0.70	1.10	0.08
Depressive disorder			2.82	<0.001	2.15	<0.01
Anxiety disorder	2.29	<0.001			2.08	<0.001
Disruptive behavior disorder	2.44	<0.001	2.24	<0.001		

*N* = 1584. Asterisks denote significant differences between childhood and adolescence risk: * *p* < 0.05, ** p < 0.01. All effects are adjusted for gender. Stress rating reflects perceived stress before the age of 12, as rated by both the adolescents and their parents. Event index reflects the weighted sum score of death of a beloved one, parental divorce, and long absence from home before the age of 12. Sexual abuse refers to unwanted sexual acts before the age of 12. All three variables were standardized to mean 0 and standard deviation 1

The largest risks of onset were found for the overall stress rating; the effects of the event index and sexual abuse were weaker and partly insignificant. Consistent with this, the effects of the stress rating remained significant after adjusting for the other childhood adversities, for all three outcomes, while the adjusted effects of sexual abuse remained only significant for depressive disorder and the effects of the event index did not reach significance for any of the outcome measures anymore (details available upon request).

As concerning the persistence of the risk associated with childhood adversities during adolescence, we found differential effects with regard to the nature of both the childhood adversities and the outcome variables. In general, the probability of a first onset of depressive disorder after childhood adversities tended to decrease over time (mean reduction in effect 56 %), while no such time-dependent decrease was found for anxiety disorders. For disruptive behavior disorders none of the time-dependent effect were significant, possibly due to the relatively low number of new onsets of disruptive behavior disorders in late adolescence (See Fig. 1), but the size and direction of the difference between childhood and adolescence risks diverged. Comparison of the different childhood adversities measures reveals that the effects of the stress rating tended to decrease, and the effects of sexual abuse to remain approximately stable over time. As the bottom half of Table 2 shows, childhood adversities were to a large extent uniquely associated with each of the outcome measures: adjusting for comorbid disorders tended to attenuate most effect sizes slightly, but the patterns remained largely comparable.

### Gender differences

We did not find any significant gender differences in the main and interaction effects under study. Gender differences in the effects of sexual abuse on disruptive behavior disorders could not be tested due to empty cells.

## Discussion

This study was conducted to test the hypothesis that the effect of exposure to childhood adversities on the risk of onset of a psychiatric disorder decreases over time, that is, was lower during adolescence than during childhood. Depressive, anxiety and disruptive behavior disorders each appeared to have their own, characteristic, pattern of associations with childhood adversities across childhood and adolescence, which was maintained after adjustment for comorbid disorders.

For depressive disorders, the overall pattern found was as expected, that is, a high excess risk during childhood, which decreased during adolescence. This pattern was by and large comparable for all measures of childhood adversities used, though not always statistically significant. For anxiety disorders, the pattern found suggested a moderately increased risk during childhood, lower than that of depressive and disruptive behavior disorders, which remained approximately stable over time. Disruptive behavior disorders took an intermediate position with regard to the effect of childhood adversities on risk of onset during childhood. The relatively few first onsets of disruptive behavior disorder during adolescence precluded reliable statements about the long-term effects of childhood adversities. The size and direction of the regression coefficients suggest that, with regard to the onset of disruptive behaviors, the long-term effects of childhood adversities depend to a larger extent on the nature of the adversity than with regard to depressive and anxiety disorders.

Childhood adversities have been associated with risk of (first) onset of psychiatric disorder before in the US National Comorbidity Survey [11] and associated studies from the same group, including the NCS Replication survey [12], NCS Adolescent Supplement [3], and World Mental Health Survey [7]. It is noteworthy that, despite substantial differences in study design and measures used, there are notable similarities between this prior research and our findings. Consistent with these studies, we found that overall ratings of the stressfulness of the environment (in the NCS-related studies operationalized as family violence) were more strongly associated with the onset of psychiatric disorders than specific events such as parental death or divorce (included in the event index). Also consistent with these studies, childhood adversities predicted anxiety disorders less strongly than depressive and disruptive behavior disorder in childhood, and the time decay was largest for depressive disorders, while there was hardly or no decay in the onset risk of anxiety disorders. The effect sizes (relative risks generally between 1.0 and 2.0) were comparable as well. There are also differences, especially with regard to the consequences of sexual abuse and the prediction of disruptive behavior disorders. Both effects tended to be smaller in our sample than in the before-mentioned studies. With regard to sexual abuse, the extremely low prevalence in our sample may have caused inaccurate effect estimates. The NCS-related studies had considerably larger samples and included sexual abuse up until the age of 18 instead of 11; differences that might underlie their larger effects. With regard to disruptive behavior disorders, these studies included attention deficit/hyperactivity disorder, which may have inflated the association with childhood adversities. But overall, we feel that the similarities between the studies outnumber the differences by far.

To the best of our knowledge, the studies referred to in the previous paragraph are the only ones that investigated childhood adversities in relation to age-dependent onset risks of multiple psychiatric disorders. A few other reports focused on depression risk in particular. Gilman et al. [36] used mother-reported childhood adversities collected at age 7 to predict the risk of depression onset during various life stages (as assessed between the age of 18 and 39) in 1,089 participants of the US National Collaborative Perinatal Project. Comparable to our findings, the effects of parental divorce, a main component of the event index used in the present study, was associated with depression onsets before the age of 15, and dropped below significance after that. Jaffee et al. [37] examined etiological differences between juvenile-onset and adult-onset cases of depressive disorder in the longitudinal Dunedin Multidisciplinary Health and Development Study. Whereas the etiology of depressive disorders with an onset before the age of 16 was characterized by perinatal problems, caretaker instability, criminality, and familial psychopathology; disorders with a later onset only had an elevated prevalence of sexual abuse, compared to non-depressed controls. Sexual abuse increased the risk of juvenile onsets as well, but not statistically significant because of little power. These findings once again confirm the notion of decreasing effects of childhood adversities on depression risk over time. Moreover, it is interesting to note that sexual abuse was the only adversity in our study that did not show a significant decay in effect from childhood to adolescence, which resembles the long-term consequences of sexual abuse reported by Jaffee and colleagues.

Our study has a number of notable strengths: a population-based sample of same-aged adolescents, a follow-up period of almost ten years, several measures of childhood adversities, from multiple informants, and information about the lifetime occurrence of DSM-IV disorders and their age of onset. Also of note is the fact that our sample was relatively young. Considering that the length of recall has been suggested to inflate estimated associations, our childhood adversity measures are probably relatively valid, compared to most other studies [12]. The combination of these factors offered unique opportunities to investigate the long-term effects of childhood adversities on later psychopathology.

A number of limitations should be accounted for when interpreting the associations found. First, exposure to childhood adversities was assessed retrospectively. Even though the length of recall was limited compared to most other studies (see above), we cannot exclude that respondents’ (imminent) psychiatric disorders during the assessment of childhood adversities influenced the ratings of the stressfulness of their childhood and perhaps also recall of specific negative events, probably in such a way that the symptoms inflated the stressfulness ratings and hence the association between childhood adversities and psychiatric disorders. The event index used is least likely to be biased by later psychopathology, because it was based on concrete, relatively objective events as reported by the parents. A second limitation is that the childhood adversities experienced by our general population sample were, on average, mild. Severe abuse or maltreatment was, to the best of our knowledge, relatively rare among the TRAILS participants, and our findings may not generalize to extremely stressful childhood conditions. Third, we focused on broad outcome domains, which could mask important differences within those categories. Particularly anxiety disorders are notoriously heterogeneous and encompass, among other things, specific phobias, which usually have an early onset and are rarely severe; social phobias, which often emerge a bit later in life and may take serious forms; and panic disorders, which tend to have a later onset and be quite severe. Taking into account differences among these specific types of anxiety could reveal different association patterns, but is beyond the scope of the present study and not feasible due to insufficient power. Fourth, we assessed psychiatric disorders at about age 19 and hence cannot make statements about the risk of psychopathology later in life.

It is remarkable that, while the risk of onset of depressive disorders associated with childhood adversities decreased over time, no such effects were found for anxiety disorders: their initial risk was lower, but remained constant over time. Tentatively, this suggests that the onset of depression is more likely to be a direct consequence of exposure to adversities than the onset of anxiety. In case of a causal stressor-disorder relationship, one expects the effect to diminish after the stressor has subsided [38]. The fact that the onset of anxiety did not show this pattern may indicate that these disorders did not result from the adversities directly, but rather from a common, unmeasured, cause, which was more stable over time than the adversities. Examples of possible common causes include, for instance, (parental) personality and genetic factors that may influence both the exposure to adversities and the outcome through gene-environment correlations (e.g., [39]). Only genetically informative study designs have the potential to tear apart individual and environmental factors thoroughly.

The declining effect of childhood adversities on the onset risk of depression may be explained by selection processes rather than a decay in risk itself. Within the individuals that are exposed to (severe) childhood adversities, the most vulnerable ones are the most likely to develop a depressive disorder. After onset, these individuals are excluded from the group who is still at risk of an incident disorder, leaving an increasingly resilient group over time. In fact, we have shown that adolescents who were exposed to childhood adversities but did not develop a depressive disorder before middle adolescence were more resilient to the depressogenic effect of stressful life events than the group of adolescents who were not exposed to childhood adversities, in whom no selection process had occurred yet [32]. From this point of view, childhood adversities can be considered a natural experiment, providing valuable information about an individual’s sensitivity or resilience to stress.

As opposed to some previous reports [27, 28], we did not find gender differences in stress-sensitivity. Although the overall risk of depression was about twice as high in girls as in boys, the relative age at onset and the relative risk associated with exposure to childhood adversities was comparable for both genders.

We used three different measures to assess childhood adversities in this study, which ranged in their degree of objectiveness from a highly subjective stressfulness rating to a much more objective index of specific events such as parental death and divorce. The stressfulness rating reflects a mixture of the occurrence of stressful events and reactions to those events. On the one hand, this measure is preferable to more objective event indices, because it encompasses all potential sources of stress, including ones that are relatively rare and therefore likely to be overlooked in event checklists. In addition, stressful events are notoriously heterogeneous with regard to their actual meaning and threat for an individual [40], and a measure incorporating the impact of events captures at least part of this heterogeneity. On the other hand, by including the stress experienced by the child in a measure, it may partly reflect a highly reactive temperament or (subthreshold) psychiatric symptoms, and hence lead to inflated estimations of the effects of stressful events on the outcome measure. In the present study, this may have happened with regard to anxiety disorders, which were predicted by the overall stress rating but not by the life events index, in particular.

To conclude, when individuals have been exposed to adversities early in life, the period that has passed since then without a psychiatric disorder contains useful information with regard to the probability that they will still develop that disorder, and this information is partly adversity-specific and disorder-specific. This has practical implications for, among other things, the development of prediction models, which should include not only information on exposure to childhood adversities, but also on mental health problems in the time since. By allowing prediction models to be updated by new information over time, and to use this information in a dynamic way, we might become better able to distinguish between individuals in need of prevention and those who are not.
